# Shear-wave elastography for muscle assessment in geriatric outpatients at increased risk of falling

**DOI:** 10.1007/s41999-025-01333-6

**Published:** 2025-10-23

**Authors:** Camilla Remuss, Josefine Oredson Krone, Ann-Kristine Weber Giger, Ditte Beck Jepsen, Karen Andersen-Ranberg, Kristoffer K. Brockhattingen

**Affiliations:** 1 Geriatric Research Unit, Department of Clinical Research, University of Southern Denmark, Odense, Denmark; 2 Department of Geriatric Medicine, Odense University Hospital, Odense, Denmark; 3 Department of Geriatrics, Odense University Hospital–Svendborg Hospital, Baagøes Alle 31, 5700 Svendborg, Denmark

**Keywords:** Shear-wave elastography, Sarcopenia, Physical performance, Ultrasound, Appendicular lean mass

## Abstract

**Purpose:**

Sarcopenia, defined by the loss of muscle strength, quantity, and quality, increases the risk of functional decline and adverse outcomes. Shear-wave elastography (SWE), a non-invasive ultrasound-based technique, quantifies muscle stiffness. However, its diagnostic value in sarcopenia remains unclear. This study investigated the relationship between rectus femoris (RF) stiffness and physical performance in geriatric outpatients with and without sarcopenia.

**Methods:**

This cross-sectional study, conducted in a geriatric outpatient clinic, included consecutive patients aged ≥ 65 years. Muscle stiffness was assessed using SWE, and muscle thickness was measured to estimate appendicular lean mass (ALM). Sarcopenia was diagnosed per EWGSOP2 criteria. Analyses included group comparisons, multivariable regression, and evaluating diagnostic accuracy using previously suggested SWE thresholds.

**Results:**

In total, 114 patients were included (mean age 80 ± 9.9 years; 65.8% female). The prevalence of sarcopenia was 20.1%. No significant difference in SWE was found between sarcopenic and non-sarcopenic participants (*p* = 0.78), nor was SWE associated with age (*ρ* = − 0.03, *p* = 0.72). Lower SWE was associated with poor physical performance (short physical performance battery ≤ 7; *β* = − 1.30, *p* = 0.025). At Q1 (4.062 kPa), the relative risk (RR) of sarcopenia was 0.83 (95% CI: 0.36–1.90); sensitivity 74%, specificity 22%. At Q3 (8.249 kPa), RR 2.33 (95% CI: 1.09–4.96); sensitivity 26%, specificity 90%. At Youden’s (8.226 kPa), RR 2.68 (95% CI: 1.31–5.47); sensitivity 30.4%, specificity 90.1%.

**Conclusion:**

Lower SWE is associated with poor physical performance. SWE is not a reliable standalone marker for sarcopenia in geriatric outpatients. Until stronger evidence emerges, assessing ALM remains preferable.

**Supplementary Information:**

The online version contains supplementary material available at 10.1007/s41999-025-01333-6.

## Key summary points

**Aims:**

To investigate the relationship between musculus rectus femoris stiffness measured by shear-wave elastography (SWE) and physical performance in geriatric outpatients with and without sarcopenia.

**Findings:**

Muscle stiffness measured with SWE did not differ significantly between sarcopenic and non-sarcopenic patients and was not associated with age. However, lower stiffness was significantly associated with poor physical performance.

**Message:**

While SWE may reflect functional status, it is not a reliable standalone diagnostic tool for sarcopenia in geriatric outpatients.

**Supplementary Information:**

The online version contains supplementary material available at 10.1007/s41999-025-01333-6.

## Introduction

Sarcopenia is a highly prevalent progressive condition, primarily affecting skeletal muscle in the older population [1]. Sarcopenia impairs muscle strength, reduces muscle mass, thus limiting physical performance. Hence, sarcopenia impairs physical and daily activities, increasing the risk of falls, fractures, prolonged hospitalization, cognitive impairment, and mortality [1, 2]. The prevalence of sarcopenia among community-dwelling middle-aged and older adults aged 50 or older varies widely, ranging from 1 to 40%, depending on the diagnostic criteria and methods used [3, 4]. A Danish study conducted in a geriatric outpatient setting found that up to one-fourth of the patients in Denmark have sarcopenia [5].

According to the algorithm proposed by the EWSGOP2[1], patients suspected of having sarcopenia must undergo the following steps: 1) Assessment of skeletal muscle strength either through handgrip strength (HGS) or chair-stand test (CST), 2) Evaluation of muscle quantity with Dual-energy X-ray absorptiometry (DXA), computed tomography (CT), magnetic resonance imaging (MRI), Bioelectrical Impedance Analysis (BIA), or ultrasound (US), 3) Assessment of physical performance using the Short Physical Performance Battery (SPPB) [1].

Sarcopenia can be slowed or even reversed through interventions focused on exercise and adequate protein intake. Given the increasing impact of sarcopenia’s adverse outcomes and the resulting financial strain on healthcare systems, early diagnosis and intervention are crucial [1, 6]. Additionally, the clinician must address underlying factors such as polypharmacy, malnutrition, and comorbidities like diabetes mellitus, atherosclerosis, and osteoporosis for optimal treatment of the condition [1, 7].

US has emerged as a recommended tool for the diagnosis of sarcopenia [1, 6, 8, 9]. It is frequently used in clinical settings and research, due to its accessibility, low cost, and non-invasive nature [10]. Compared to CT, MRI, DXA, and BIA, conventional US offers significant advantages such as portability, immediate results, independence from hydration status, no radiation, and reduces the need for multiple outpatient appointments, enhancing patient compliance and comfort [1, 6]. In addition, US is a patient-centered method that can be performed bedside by the attending physician. A notable extension of conventional US is shear-wave elastography (SWE) [10], which quantifies tissue stiffness by measuring the velocity of shear wave generated within the muscle [11]. SWE can detect changes in anatomical characteristics of the muscle, such as fat infiltration and fibrosis, which are associated with reduced muscle function [12]. Thus, it is believed that increased muscle stiffness, as measured via SWE, is associated with reduced functional muscle mass. However, recent studies have shown contradictory results regarding this hypothesis with some muscle showing no association between SWE and physical functioning [13–15].

SWE is well-established for assessing hepatic stiffness and characterizing breast and thyroid tumors [11, 16, 17] and has shown reliability in measuring muscle stiffness [6, 12, 17, 18].

However, the use of SWE to assess anatomical characteristics of the muscle remains largely unstandardized, highlighting the need for further research. Although Bastijns et al. have proposed evidence-based guidelines for SWE measurement of muscle, reference values on a grade scale have yet to be established [6]. In this context, US-based modalities such as SWE offer promising potential for practical, bedside application, thereby supporting wider clinical adoption and enabling more accessible assessment of anatomical characteristics of the muscle in everyday practice. The overall aim of this cross-sectional study was to investigate the relationship between muscle stiffness of the rectus femoris (RF), assessed using US shear-wave elastography, and physical performance in geriatric outpatients aged ≥ 65 years, with and without sarcopenia, referred to a Falls Clinic. Further aims were to investigate the association between SWE and age, and to evaluate the accuracy of SWE for identifying sarcopenia.

## Materials and methods

### Setting and study population

This cross-sectional study was conducted at the Falls Clinic, Odense University Hospital–Svendborg, Denmark, from October 2, 2024, to December 18, 2024. All patients aged ≥ 65 years were invited to participate during their first visit to the Falls Clinic. Participants were informed about the study and provided written consent prior to inclusion. Those with impaired cognitive function, such as moderate to severe dementia, were excluded prior to enrolment. Those with missing US assessment and/or physical test were initially included but subsequently excluded from the final analyses.

### Sample-size calculation

Due to the exploratory nature of this study, a power calculation was conducted to estimate the prevalence of sarcopenia within the study population. The expected prevalence of sarcopenia was set to 20%, based on a prior study conducted on a similar population that reported a prevalence of sarcopenia of 26% [5]. Power analysis was performed with a power of 80% and a significance level of 5%, yielding a sample size of 114 participants. This approach was chosen to ensure an adequate number of participants with sarcopenia for exploratory analyses.

### Clinical characteristics

#### Body mass index

Height (meters, m) was assessed either without shoes to the nearest 0.1 cm or with shoes, where 2 cm was deducted from the measured height. Weight (kilograms, kg) was measured using a calibrated Seca scale, with participants wearing normal indoor clothing to the nearest 0.1 kg. A – 1 kg adjustment was made for the clothing. Body mass index (BMI) was calculated as weight/height^2^ (kg/m^2^).

#### Falls

A fall was defined as an unexpected event resulting in the person coming to rest on the ground or a lower level [19]. The number of patient-reported falls within 12 months was recorded.

##### Comorbidity

Data on comorbidities were retrieved from the patients’ medical records. The degree of comorbidity was assessed using the Charlson Comorbidity Index (CCI) [20], a widely used and validated tool for estimating mortality risk. Its predictive value has been demonstrated in multiple populations and it also shows good inter-rater reliability [21]. A score ≥ 3 indicates a significant increase in mortality (~ 50%) compared to the background population [20].

##### Clinical frailty scale

Clinical Frailty scale (CFS) was retrieved from the patients’ medical records on the day of study inclusion, which coincided with the day of study assessments. The CFS is a validated tool used to measure frailty (1 = fit – 9 = bedridden), which can be utilized in medical decision-making [22]. A score ≥ 5 indicates frailty, with increasing scores indicating greater frailty [23]. Each patient’s level of frailty was assessed by consensus agreement within the geriatric team. If members of the geriatric team performing the falls evaluation reported different CFS scores, the score of the treating physician was chosen.

##### Sarcopenia

Sarcopenia was assessed using the EWGSOP2 criteria:

- *Muscle strength*: HGS < 16 kg and < 27 kg for women and men, respectively, and if the 5-time CST took more than 15 s to complete [1]. Participants with a SPPB score in the 5-time CST < 2 were classified as having sarcopenia
- *Muscle quantity*: ALM < 15 kg and < 20 kg for women and men, respectively [1]. ALM is calculated using the method proposed by Barbosa-Silva et al. [24], using US measurements and the following calculation:
  - $$2.39*sex+15.14*height+0.29*arm length+1.93*muscle tichness arm+0.87* muscle thickness theigh-23.7$$
- *Muscle stiffness* was assessed by SWE using an approach recommended from the current literature as described by Bastijns et al. and Okyar Baş et al. [6, 12].

### Physical performance assessments

The physical performance was evaluated using the 30-s CST, HGST, and SPPB, including the usual gait speed test, standing balance, and 5-times CST.

All the physical performance assessments were supervised by the outpatient clinic physiotherapists or trained medical students (study personnel).

#### SPPB

SPPB was performed as originally described and validated by Guralnik et al., using the standard scoring system (0–4 per subtest; maximum 12 points) [25]. It is a validated method with a strong correlation to lower extremity function [26].

The SPPB score serves as a measure of an individual’s physical performance status and functional capacity in activities of daily living (ADL) [27]. A lower SPPB score is associated with an increased risk of falls and a higher all-cause mortality rate [28, 29]. A score ≤ 6 indicates high risk for fall and all-cause mortality, poor physical and ADL function; 7–9 indicates increased risk for fall and all-cause mortality, and 10–12 indicates low risk, good physical and ADL function [27–29]. Further, a score ≤ 8 indicates poor physical health [1].

#### Muscle strength

HGS was measured using a calibrated Baseline BIMS Digital Hand Dynamometer in kilograms. The measurements were performed using the participants’ dominant hand in an upright seated position, with the elbow held near the body, bent at a 90-degree angle supported by a horizontal surface, and the hand positioned in a neutral position. The measurement was repeated three times, and the best was used for analysis. The Baseline BIMS Digital Hand Dynamometer demonstrated strong correlation, excellent inter-instrument reliability, and low measurement error with the Jamar + Hand Dynamometer, which is the gold standard for HGS measurement [30], allowing for interchangeable use [31].

### Ultrasound assessment

The assessment was performed using a CE-certified Siemens ACUSON S3000 US machine with a 9L4 probe (linear transducer), set to the B-mode musculoskeletal setting. The scanning personnel were trained by a US expert with experience from over 1000 completed scans, following the established protocol. Data acquisition was performed under ongoing supervision.

#### Muscle thickness

Muscle thickness was measured on both the dominant upper arm and leg using the protocol from the Sarcopenia through Ultrasound working group (SARCUS) [9]. If a patient was unsure of which leg was their dominant leg, they were asked, “Which leg would they kick a ball with?” and this leg was chosen as dominant.

The probe was held at a 90-degree angle to the skin, with minimal pressure in a transversal orientation. On the upper arm, we measured at the distal 2/2 between the acromion and the upper border of the olecranon with the arm in a relaxed supine position [9]. On the leg, we measured at the middle between the spina iliac anterior superior and the upper border of the patella in a supine and relaxed position [8, 9]. All measurements were performed 3 times with a new placement of the probe each time. The data on the muscle thickness (MT) were used in the ALM calculation.

#### Shear-wave elastography

SWE was performed on the dominant leg at the same position as the MT measurement, with the probe in a longitudinal orientation to obtain measurements in a sagittal/longitudinal image. SWE operates by applying an acoustic radiation force impulse (ARFI) to generate shear waves, which travel through the tissue. The velocity of these waves is detected by the US transducer, providing measurements in shear-wave velocity (SWV, m/s), which are subsequently converted into tissue stiffness (SWE, kPa) using Young’s modulus. This stiffness measurement, referred to as SWE, represents the tendency of a tissue to stretch and deform [6].

To minimize potential measurement errors, plenty of gel was applied, and care was taken to avoid compression or deformation of the superficial endomysium. Participants refrained from physical activity for 5–30 min prior to assessment [9, 17, 32]. The measurement was repeated three times, repositioning the region of interest (ROI) each time to ensure consistent ROI size (1 cm × 1 cm) and placement away from the muscle fascia, in the upper half of the muscle to prevent impact on elasticity [32]. There were no restrictions on measurement depth [32]. SWE has demonstrated excellent inter-rater and intra-rater reliability, as well as good to very good inter-day observer reliability when measuring the RF [17].

### Statistics

Statistical analyses were performed using R (version 2024.12.1 + 563). The normality of variables was assessed using the Shapiro–Wilk test. Normally distributed data were presented as mean ± standard deviation (SD), while non-normally distributed data were presented as median with interquartile range (IQR). Comparison between participants with and without sarcopenia was assessed using a t–test if data were normally distributed or a Mann–Whitney U test for non-normally distributed data.

The Relative Risk (RR) of being diagnosed with sarcopenia based on SWE was estimated using a 2 × 2 contingency table. The optimal SWE threshold for classification was initially identified through ROC curve analysis, using Youden’s index to determine the best cut-off value. However, due to the high specificity (90%) and low sensitivity (30%) of this threshold, a post hoc analysis was performed to explore the first (Q1) and third (Q3) quartiles of SWE as thresholds instead. Besides RR, we also investigated sensitivity, specificity, and overall accuracy at each threshold.

The association between SWE and age was investigated using Spearman’s correlation coefficients, due to the nature of the SWE values. Correlation coefficients (*ρ*-values) range from − 1 to + 1, indicating the strength and direction of the association.

Multivariable linear regression was conducted with SWE (kPa) as the dependent variable. Covariates included HGS, CCI, CFS, SPPB, number of falls, and sex. Age was not included in the model. Covariates were categorized using clinically relevant thresholds: HGS was dichotomized according to EWGSOP2 criteria (< 16 kg for females and < 27 kg for males) [1]; CCI was grouped as ≤ 2 vs. ≥ 3 due to increased mortality risk above this threshold [1]; CFS was categorized as ≤ 4 vs. ≥ 5 to distinguish between non-frail and frail individuals; SPPB was grouped as ≤ 7 vs. ≥ 8, reflecting poor versus good physical function; and falls were categorized as ≤ 1 vs. ≥ 2, based on the recognition that fall history is an important risk factor in fall prevention [33].

Intraclass correlation coefficient (ICC) analysis was conducted to assess agreement between raters.

Statistical assistance was provided by a professional statistician in selecting the model structure and interpreting the results. A *P*-value < 0.05 was considered statistically significant.

## Results

### Population characteristics

**Fig. 1:**
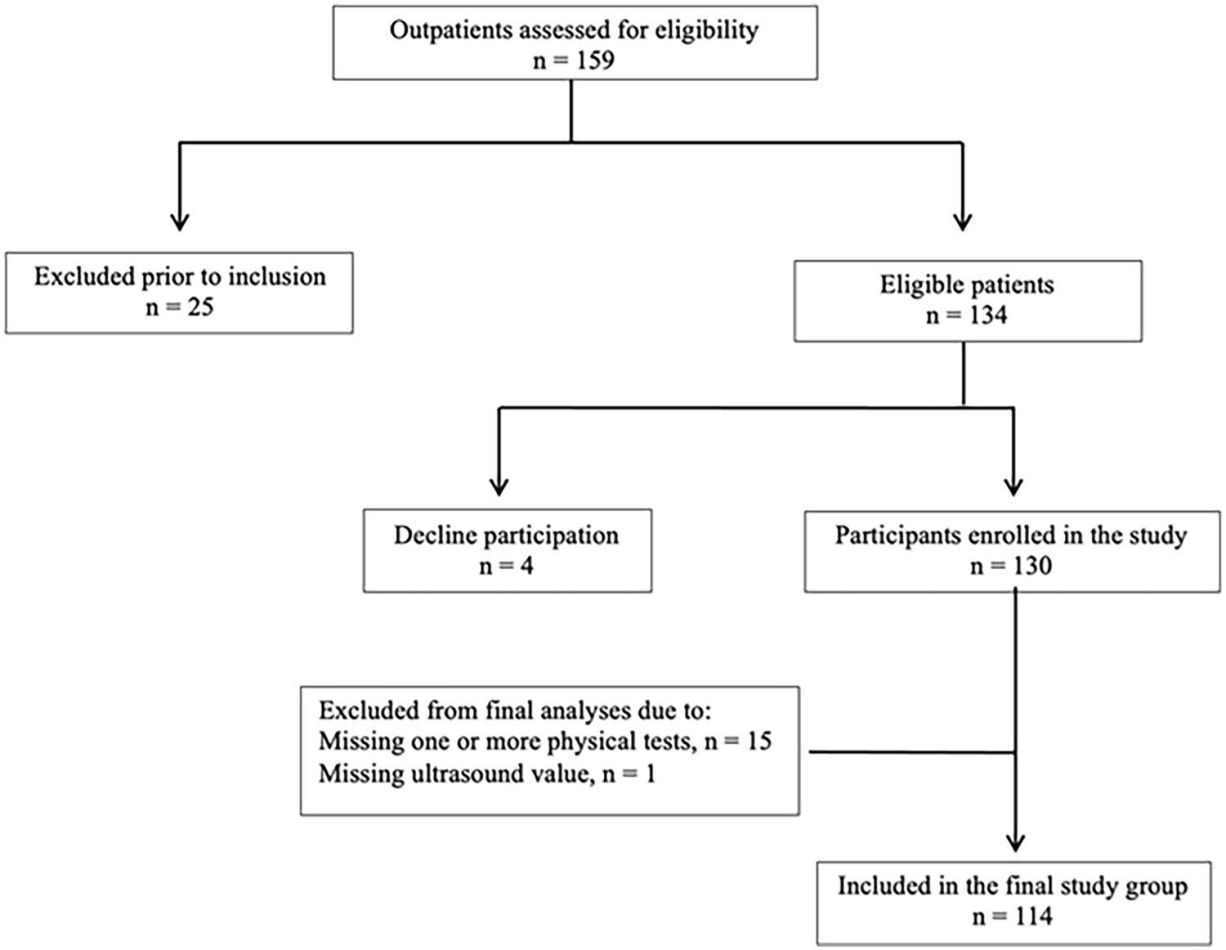
Of the 159 outpatients assessed for eligibility, 25 were excluded prior to inclusion. Among the 134 eligible patients, 4 declined participations resulting in 130 participants enrolled in the study. Of these, 17 were excluded from the final analysis due to missing data (15 due to incomplete physical performance tests and 1 due to missing ultrasound values), leaving 114 participants in the final study group

A total of 130 patients agreed to participate (participation rate 97%, *n* = 126) and underwent physical, clinical, physical performance, and ultrasound assessments. Of these, 114 participants with complete datasets were included in the statistical analyses; for details, see Fig. 1.

Of these, 65.8% were women. Participants were aged from 65 to 95 years with a mean age of 80 years (SD: 7, IQR: 75–85). BMI ranged from 15.24 to 42.67, with a median of 26.3 (IQR: 22.0–29.4).

The median number of falls within the last 12 months was 3 (IQR: 1–6). The CFS and CCI had median values of 5 (IQR: 4–6) and 4 (IQR: 3–5), respectively.

The mean HGS was 29.07 kg (SD: 7.89) for males and 19.33 kg (SD: 4.67) for females. The SPPB had a median score of 7 (IQR: 4–9), and the 30 s CST had a median of 7 repetitions (IQR: 0–10). Mean MT was 2.03 cm (SD: 0.52) in the arm and 2.92 cm (SD: 0.81) in the thigh. Median ALM was 23.98 kg (IQR: 21.91–25.15) for males and 16.88 kg (IQR: 15.26–18.94) for females. Median SWV was 1.27 m/s (IQR: 1.14–3.33), and the median SWE was 5.22 kPa (IQR: 4.21–6.45). The overall prevalence of sarcopenia in the cohort was 20.1% (23/114).

A full overview of baseline characteristics is presented in Table 1.

**Table 1:** Baseline characteristics of the included participants

*N* = 114	Mean	SD	Median	IQR
Clinical characteristic				
Age (years)	80	7		
BMI (kg/m^2^)			26.3	22.0–29.4
Fall (within the last 12 months)			3	1–6
CCI (0–37 points)			5	4–6
CFS (1–9 point)			4	3–5
Ultrasound assessment				
SWV (m/s)			1.27	1.14–1.43
SWE (kPa)			5.22	4.21–6.45
MT arm (cm)	2.03	0.52		
MT thigh (cm)	2.92	0.81		
ALM female (kg)			16.88	15.26–18.94
ALM male (kg)			23.98	21.91–25.15
Physical performance assessment				
30-s CST			7	0–10
HGS Female (kg)	19.3	4.7		
HGS male (kg)	29.1	7.9		
SPPB (0–12 point)			7	4–9
Standing balance test (0–4 point)			3	1–4
3 m usual gait-speed test (0–4 point)			3	2–4
5-time CST (0–4 point)			1	0–2

Data are presented as mean ± SD for normally distributed variables, median (IQR) for non-normally distributed variables

*ALM* Appendicular lean mass, *BMI* Body mass index, *CCI* Charlson Comorbidity index, *CFS* Clinical frailty scale, *CI* Confidence interval, *CST* Chair-stand test, *HGS* Handgrip strength, *IQR* Interquartile range, *MT* Muscle thickness, *N* Number, *SD* Standard deviation, *SPPB* Short physical performance battery, *SWE* Shear-wave elastography, *SWV* Shear-wave velocity

The 16 excluded participants had incomplete data, primarily due to missing ultrasound or performance assessments. Of the excluded participants, 19% had sarcopenia, 50% were women, and the mean age was 80 years. Overall, the baseline characteristics of those excluded did not differ significantly from those included.

Baseline characteristics and between-group comparisons of included and excluded participants are presented in Supplementary Table 1 located in the supplementary material.

### Comparison of baseline characteristics between those with and without sarcopenia

The baseline characteristics were compared between the included participants stratified by sarcopenia status. No significant differences were observed between the groups in terms of age, sex, SWV, SWE, CCI, or the number of falls within the past 12 months. Participants with sarcopenia had significantly lower physical performance measures, including HGS in both males and females, SPPB scores, and the 30 s CST, compared to those without sarcopenia. Additionally, they had significantly lower BMI and ALM in both sexes, as well as higher CFS scores. A complete overview of baseline characteristics and comparison analyses is presented in Table 2.

**Table 2:**
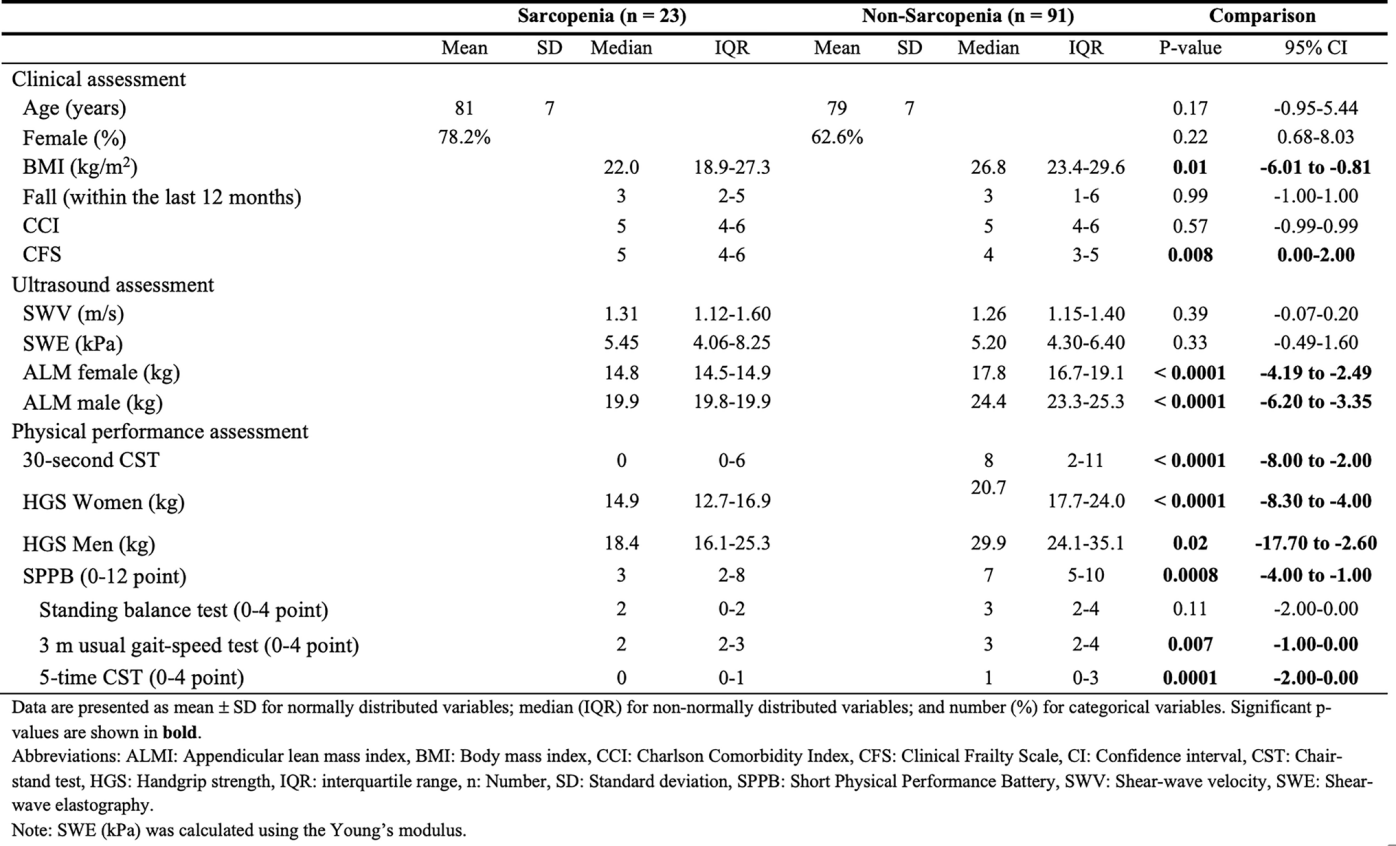
Baseline characteristles of the included participants stratified by sarcopenia status

Data are presented as mean ± SD for normally distributed variables, median (IQR) for non-normally distributed variables, and number (%) for categorical variables

SWE (kPa) was calculated using the Young's modalus

*ALMI* Appendicular lean mass index, *BMI* Body mass index, *CCI* Charison comorbidity index, *CFS* Clinical frailty scale, *CI* Confidence interval, *CST* Chair stand test, *HGS* Handgrip strength, *IQR* Interquartile range, *n* Number, *SD* Standard deviation, *SPPB* Short physical performance battery, *SWV* Shear-wave velocity, *SWE* Shear-wave elastography

Significant p values are shown in bold

### Diagnostic accuracy in using SWE to diagnose sarcopenia

**Fig. 2:**
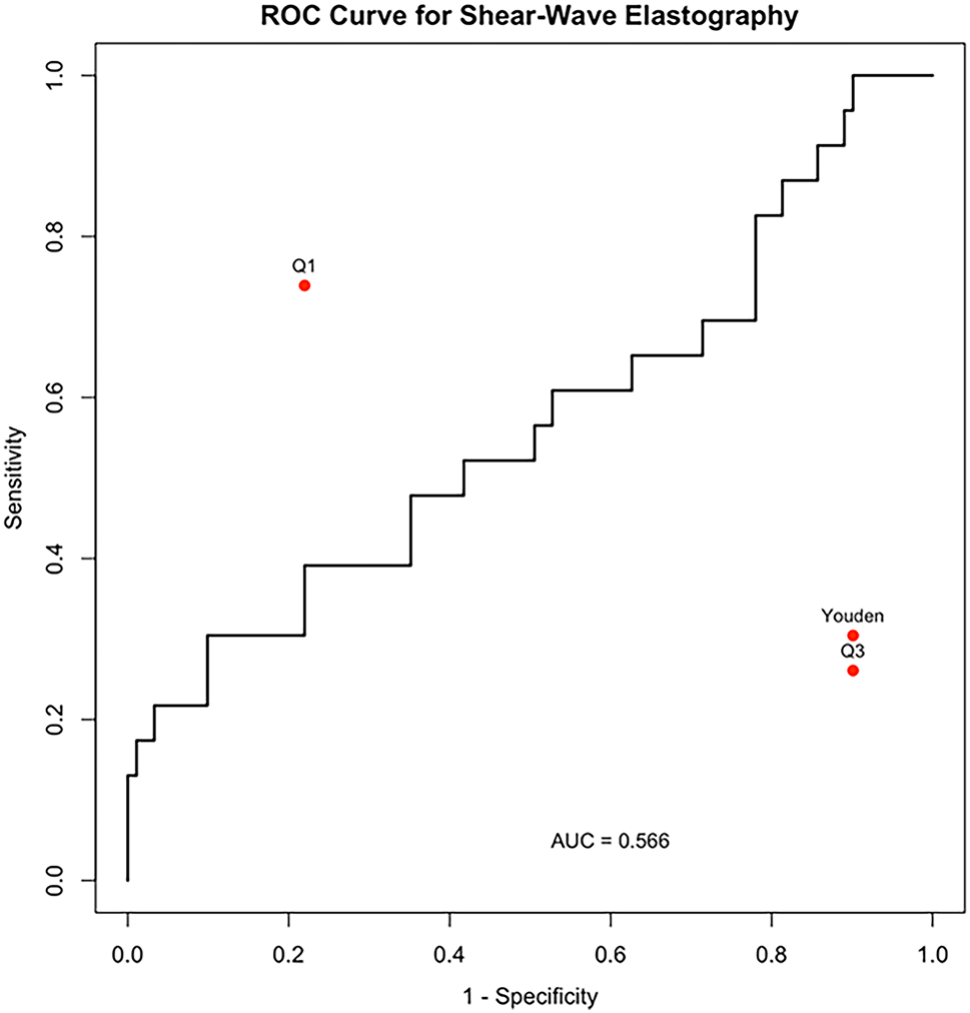
ROC curve illustrating the diagnostic performance of shear-wave elastography (SWE) for identifying sarcopenia. The cu1-e plots sensitivity versus 1—specificity across all possible SWE thresholds, with an area under the curve (AUC) of 0.566. Three thresholds are highlighted: Q1 (4.06 kPa), Youden’s index (8.23 kPa), and Q3 (8.25 kPa). At the Q1 threshold, sensitivity was high (74%) but specificity was low (22%), while the Q3 threshold showed high specificity (90%) but low sensitivity (26%). The Youden threshold provided a balance with moderate sensitivity and specificity

To assess the clinical relevance of SWE, the relative risk of sarcopenia was calculated for participants with different SWE values. Thresholds were based on Youden’s index, first (Q1) and third (Q3) quartile of the SWE, illustrated in Fig. 2. Using the Q1 threshold of 4.062 kPa, the prevalence of sarcopenia was 19% (17/88) among participants with SWE ≥ 4.062 kPa, and 23% (6/26) among participants with SWE < 4.062 kPa, corresponding to a RR of 0.83 (95% CI: 0.36–1.90). At this threshold, the sensitivity was 74%, specificity 22%, resulting in an overall accuracy of 32.5%. When applying the Q3 threshold of 8.249 kPa, 40% (6/15) of participants with SWE values ≥ 8.249 kPa had sarcopenia, and 17% (17/99) in the group with SWE < 8.249 kPa. The corresponding RR in this case was 2.33 (95% CI: 1.09–4.96). At this threshold, sensitivity was 26%, specificity 90%, resulting in an overall accuracy of 77.2%. Additionally, Youden’s index identified 8.226 kPa as the optimal threshold. At this threshold, sarcopenia prevalence was 43.8% (7/16) in the group with SWE ≥ 8.226 kPa, and 16.3% (16/98) in the group with SWE < 8.249. The corresponding RR was 2.68 (95% CI: 1.31–5.47). Sensitivity was 30.4% and specificity was 90.1%, resulting in an overall accuracy of 78.1%.

### Association between SWE and age

We explored whether SWE values varied with chronological age. No significant correlation was observed between age and SWE (*ρ* = 0.16, *p* = 0.1).

### SWE and its association with clinical and physical parameters

To identify factors associated with muscle stiffness, a multivariate linear regression analysis was conducted with SWE (kPa) as the dependent variable. Due to non-normally distributed residuals, robust standard errors were applied. The analysis showed that participants with low physical performance (SPPB ≤ 7) had significantly lower SWE (kPa) compared to those with high performance (SPPB ≥ 8) (*β* = − 1.30, *p* = 0.025). A trend toward higher SWE was observed in participants with higher frailty scores (CFS 5–9) (*β* = 1.61, *p* = 0.095). No significant associations were identified for HGS, CST, CCI, number of falls, or sex.

### Interclass correlation

To ensure measurement reliability, an intraclass correlation coefficient (ICC) was calculated between an ultrasound expert (with > 1000 completed scans) and two trained medical students. The ICC yielded a value of 0.98.

## Discussion

In this cohort of 114 geriatric outpatients aged ≥ 65 years referred to a Falls Clinic, the overall prevalence of sarcopenia was 20.1%. The sample size fulfilled the requirements of the prior power calculation, which had been based on an expected prevalence of sarcopenia in this population. Of the 130 patients who initially agreed to participate, 16 were excluded from the analyses due to missing data. These missing data were primarily due to logistical constraints in the daily clinical workflow, rather than participants’ inability to complete the assessments. Notably, the excluded participants had similar baseline characteristics to those included in the analyses, with no significant differences between groups (see Supplementary Table 1).

Participants with sarcopenia had significantly lower physical performance (e.g., HGS, SPPB, 30-s CST), lower BMI and ALM (stratified by sex), and were frailer (higher CFS scores) than those without sarcopenia. No significant differences were observed between groups regarding age, sex, number of falls, CCI, or SWE measurements. SWE showed no significant association with chronological age, suggesting that muscle stiffness of the rectus femoris does not vary substantially among individuals aged 65 years and older in our cohort. Three different RR were identified depending on the chosen threshold; however, none of the cut-offs showed a robust association with sarcopenia. In the multivariable regression analysis, a significant association was observed between lower SWE (kPa) values and poor physical performance (SPPB ≤ 7).

The observed prevalence of sarcopenia (20.1%) is in line with previous studies, such as Christensen et al. (26%) [5] and Nielsen et al. (14.2%) [34], both conducted in similar cohorts. Differences in prevalence may be attributed to variations in diagnostic criteria (EWGSOP vs. EWSGOP2), comorbidities, and assessment methods such as DXA and BIA. The significantly lower physical performance, BMI, and ALM in the sarcopenic participants were consistent with expectations, based on the diagnostic criteria for sarcopenia, which include assessments of muscle mass and physical function [1].

No significant association was found between the RF muscle stiffness and age in this cohort, indicating that muscle stiffness measured by SWE does not appear to vary substantially with age in this cohort. The existing literature on this topic remains inconclusive. For instance, Saito et al. [35] investigated RF muscle stiffness in healthy young female students and healthy older community-dwelling females, reporting increased muscle stiffness with age. In contrast, Akagi et al. [36] and Alfurahi et al. [37] examined both male and female participants and found the opposite: older individuals exhibited decreased muscle stiffness compared to younger individuals. These conflicting findings may stem from varying definitions of “older”, differences in physical activity levels, and a lack of consensus regarding age-related changes in muscle properties [6, 38].

Further studies across diverse age groups are needed to clarify the effect of age on muscle stiffness.

Applying a lower SWE threshold (≥ Q1; 4.062 kPa) did not significantly differentiate sarcopenia risk (RR = 0.83, 95% CI: 0.36–1.90), suggesting a non-significant trend toward a lower risk of sarcopenia among individuals with higher SWE values. In contrast, a higher threshold (≥ Q3; 8.249 kPa) was associated with a significantly greater risk of sarcopenia with higher SWE values (RR = 2.33, 95% CI: 1.09–4.96). Youden’s index threshold (8.226 kPa) with an RR of 2.68 (95% CI: 1.31–5.47), demonstrates similar performance to the Q3 threshold, with slightly improved balance between true positive and false positive classifications.

Contrary to prior assumptions, no significant differences in SWE measurements were observed between sarcopenic and non-sarcopenic participants, even though prior research has promoted SWE as a promising method to assess anatomical characteristics of the muscle [8, 10]. This discrepancy with studies such as Wang et al. [39], who reported higher SWV in non-sarcopenic individuals, may reflect variations in measurement techniques, target muscles, and population characteristics. For example, the cohort in Wang et al. had a lower mean age (approximately 62 years vs. 80 years), a slightly different sex distribution (59% female vs. 66% female), and notably thinner rectus femoris MT (approximately 1.5 cm vs. 2.9 cm) compared to the present study. The heterogeneity in SWE technology and the lack of standardized protocols may contribute to inconsistent results across studies.

Studies by Okyar Baş et al. [12] and Bastjans et al. [6] both highlight the need for further research to establish an optimal cut-off value, as it remains undetermined. The diagnostic accuracy varied by SWE threshold. A lower threshold set by the first quartile (Q1, SWE ≥ 4.062 kPa) provided high sensitivity but poor specificity, resulting in numerous false positives (n = 71). In contrast, the higher threshold set by the third quartile (Q3, SWE ≥ 8.249 kPa) offered improved specificity but reduced sensitivity. Accurate diagnosis in older adults is challenging; both over- and underdiagnosis carry unwanted risks [40]. Overdiagnosis can lead to unnecessary stress, additional testing, potential overtreatment, and strain on healthcare systems [40, 41]. Underdiagnosing, on the other hand, risks missing timely and necessary intervention, ultimately worsening health outcomes.

Therefore, selecting an appropriate threshold requires a careful balance, and further studies are needed to refine these thresholds and enhance diagnostic accuracy.

In the multivariate regression analysis, a significant association was found between lower SWE (kPa) values and poor physical performance (SPPB ≤ 7), suggesting a link between muscle stiffness and functional decline. These findings were somewhat unexpected, as previous studies have suggested that SWE may be a useful tool for diagnosing sarcopenia [6], given its ability to assess anatomical characteristics of the muscle [8, 10], a key component of the diagnosis, alongside physical performance and muscle mass [1]. However, in this study, SWE was associated only with physical performance. This highlights the complexity of the current EWGSOP2 definition, where physical performance alone is not sufficient for diagnosis [1]. Although SWE may capture aspects of anatomical characteristics of the muscle relevant to functional status, the findings suggest that other components, such as muscle quantity, may currently weigh more heavily in the current diagnostic process of sarcopenia. Should a further diagnostic framework place greater emphasis on physical performance, SWE could become a more relevant assessment modality.

Nonetheless, the results of this study do not support a clear advantage of using SWE over conventional US. Moreover, SWE has practical limitations, such as relying on more complex technology and lacking suitability for point-of-care testing (POCT). Some associations were contrary to expectations, and further studies with larger samples and broader patient representation—including men and younger individuals—are needed. A survey by Davis et al. [11], including 163 musculoskeletal radiologists in the United States, found that although 89.5% had access to conventional US, only 11.8% had SWE for muscle assessment available. Given these limitations, continued use of conventional US is suggested to measure MT and to calculate ALM as a more practical and accessible approach. Based on the findings of this study and the availability of US in clinical practice, SWE cannot currently be recommended as a standalone tool for sarcopenia assessment in clinical practice.

Consistent with our results, Fadiloglu et al. (earlier this year) also reported no association between shear-wave elastography (SWE) and frailty [13]. Notably, their study did find a significant association between muscle thickness (MT) and SWE. This is clinically relevant because MT can be measured with handheld ultrasound in point-of-care settings, whereas SWE typically requires dedicated equipment—suggesting MT may be a pragmatic surrogate when SWE is impractical.

Several limitations should be noted. The cross-sectional design limits the ability to establish a causal relationship between muscle stiffness and sarcopenia. In addition, ALM as estimated by ultrasound has not been established as a recommended measurement of ALM. This might reduce comparability to other studies and is a clear limitation. Additionally, the relatively small number of male participants may have reduced the statistical power to detect sex-specific associations, increasing the risk of Type II errors. Fall history was self-reported, which may introduce some recall bias; however, this was not a primary outcome. Furthermore, results of the post-hoc analysis of share wave elastography for cut-off points for sarcopenia classification are sample specific and, as such, have limited generalizability. This limits the external validity of this study, although the results of the post-hoc analysis did not add any value to the results.

Despite these limitations, the study has several notable strengths. The sample represents a relatively large cohort of geriatric outpatients—a population that is often challenging to recruit due to age-related health and mobility limitations [42]. Remarkably, only four patients declined participation, which minimizes the risk of selection bias and strengthens the generalizability within this population. Importantly, all eligible patients were included regardless of comorbidity burden, resulting in a more representative, real-world sample rather than a selectively healthy subgroup. The ICC yielded a value of 0.98, which is considered excellent [43], supporting the robustness of the measurements. This aligns with previous findings by Cui et al. [16], suggesting a relatively short learning curve for both US and SWE methods.

## Conclusions

In conclusion, no significant differences in RF muscle stiffness, assessed by SWE, were found between geriatric outpatients with and without sarcopenia. In this cohort, RF muscle stiffness showed no association with chronological age, indicating limited variation among individuals aged ≥ 65 years with comparable clinical profiles. Although lower SWE values were associated with poorer physical performance, the diagnostic utility of SWE appears limited due to low sensitivity at clinically relevant thresholds and the absence of validated cut-off values. Until further evidence emerges, ALM, measured via conventional US, remains the preferred parameter for sarcopenia assessment in this population.

## Supplementary Information

Below is the link to the electronic supplementary material.

Supplementary file1 (DOCX 307 KB)

## Data Availability

Qualified researchers from an appropriate institution may request access to individual participant data that underlie the results reported in this article, after de-identification (text, tables, figures, and appendices). Upon approval of a data sharing request by the regional legal institution, information necessary to address the research question will be provided under the terms of a signed data sharing agreement. Requests should be submitted to: kristoffer.k.brockhattingen@rsyd.dk.

## Abbreviations

ALM: Appendicular lean mass
ARFI: Acoustic radiation force impulse
BIA: Bioelectrical impedance analysis
BMI: Body mass index
CCI: Charlson comorbidity index
CFS: Clinical frailty scale
CI: Confidence interval
cm: Centimeter
CST: Chair-stand test
CT: Computed tomography
DXA: Dual-energy X-ray absorptiometry
EWGSOP: European working group of sarcopenia in older people
EWGSOP2: European working group of sarcopenia in older people 2
HGS: Handgrip strength
ICC: Intraclass correlation coefficient
IQR: Interquartile range
kg: Kilograms
kPa: Kilopascal
m: Meter
MRI: Magnetic resonance imaging
MT: Muscle thickness
N: Number
OPEN: Odense patient data explorative network
Q1: 25Th quartile
Q3: 75Th quartile
RF: Rectus femoris
ROI: Region of interest
RR: Relative risk
s: Seconds
SARCUS: The sarcopenia through ultrasound working group
SD: Standard deviation
SPPB: Short physical performance battery
SWE: Shear-wave elastography
SWV: Shear-wave velocity
US: Ultrasound

## References

[1] Cruz-Jentoft AJ, Bahat G, Bauer J, Boirie Y, Bruyère O, Cederholm T, Cooper C, Landi F, Rolland Y, Sayer AA, Schneider SM, Sieber CC, Topinkova E, Vandewoude M, Visser M, Zamboni M (2019) Sarcopenia: revised European consensus on definition and diagnosis. Age Ageing 48(1):16–31. 10.1093/ageing/afy169 30312372 10.1093/ageing/afy169 PMC6322506

[2] Yuan S, Larsson SC (2023) Epidemiology of sarcopenia: prevalence, risk factors, and consequences. Metabolism 144:155533. 10.1016/j.metabol.2023.155533 36907247 10.1016/j.metabol.2023.155533

[3] Cruz-Jentoft AJ, Landi F, Schneider SM, Zúñiga C, Arai H, Boirie Y, Chen LK, Fielding RA, Martin FC, Michel JP, Sieber C, Stout JR, Studenski SA, Vellas B, Woo J, Zamboni M, Cederholm T (2014) Prevalence of and interventions for sarcopenia in ageing adults: a systematic review. report of the international sarcopenia Initiative (EWGSOP and IWGS). Age Ageing 43(6):748–759. 10.1093/ageing/afu115 25241753 10.1093/ageing/afu115 PMC4204661

[4] Mayhew AJ, Amog K, Phillips S, Parise G, McNicholas PD, de Souza RJ, Thabane L, Raina P (2018) The prevalence of sarcopenia in community-dwelling older adults, an exploration of differences between studies and within definitions: a systematic review and meta-analyses. Age Ageing 48(1):48–56. 10.1093/ageing/afy106 10.1093/ageing/afy106 30052707

[5] Christensen MG, Piper KS, Dreier R, Suetta C, Andersen HE (2018) Prevalence of sarcopenia in a Danish geriatric out-patient population. Danish Med J 65(6):A5485 29886880

[6] Bastijns S, De Cock AM, Vandewoude M, Perkisas S (2020) Usability and pitfalls of shear-wave elastography for evaluation of muscle quality and its potential in assessing sarcopenia: a review. Ultrasound Med Biol 46(11):2891–2907. 10.1016/j.ultrasmedbio.2020.06.023 32843232 10.1016/j.ultrasmedbio.2020.06.023

[7] Deutz NE, Bauer JM, Barazzoni R, Biolo G, Boirie Y, Bosy-Westphal A, Cederholm T, Cruz-Jentoft A, Krznariç Z, Nair KS, Singer P, Teta D, Tipton K, Calder PC (2014) Protein intake and exercise for optimal muscle function with aging: recommendations from the ESPEN expert group. Clin Nutr 33(6):929–936. 10.1016/j.clnu.2014.04.007 24814383 10.1016/j.clnu.2014.04.007 PMC4208946

[8] Yi J, Cha JG, Hahn S (2023) Comparison of shear wave elastography with gray-scale USG and CT for quantitative evaluation of rectus femoris muscle. J Clin Ultrasound 51(4):703–710. 10.1002/jcu.23435 36710597 10.1002/jcu.23435

[9] Perkisas S, Bastijns S, Baudry S, Bauer J, Beaudart C, Beckwée D, Cruz-Jentoft A, Gasowski J, Hobbelen H, Jager-Wittenaar H, Kasiukiewicz A, Landi F, Małek M, Marco E, Martone AM, de Miguel AM, Piotrowicz K, Sanchez E, Sanchez-Rodriguez D, Scafoglieri A, Vandewoude M, Verhoeven V, Wojszel ZB, De Cock A-M (2021) Application of ultrasound for muscle assessment in sarcopenia: 2020 SARCUS update. Eur Geriatr Med 12(1):45–59. 10.1007/s41999-020-00433-9 33387359 10.1007/s41999-020-00433-9

[10] Taljanovic MS, Gimber LH, Becker GW, Latt LD, Klauser AS, Melville DM, Gao L, Witte RS (2017) Shear-wave elastography: basic physics and musculoskeletal applications. Radiographics 37(3):855–870. 10.1148/rg.2017160116 28493799 10.1148/rg.2017160116 PMC5452887

[11] Davis LC, Baumer TG, Bey MJ, Holsbeeck MV (2019) Clinical utilization of shear wave elastography in the musculoskeletal system. Ultrasonography 38(1):2–12. 10.14366/usg.18039 30343557 10.14366/usg.18039 PMC6323314

[12] Okyar Baş A, Baş H, Ceylan S, Güner Oytun M, Koca M, Hafızoğlu M, Şahiner Z, Öztürk Y, Balcı C, Doğu BB, Cankurtaran M, Halil MG (2023) Changes in muscle quality identified by shear-wave elastography and association with sarcopenia. JPEN J Parenter Enteral Nutr 47(2):253–264. 10.1002/jpen.2457 36227071 10.1002/jpen.2457

[13] Fadiloglu A, Cataltepe E, Ceker E, Allahverdiyeva S, Samadli S, Sendur HN, Güngör F, Varan HD (2025) Comparison of rectus femoris muscle shear wave elastography and thickness on evaluation of frailty. Eur Geriatr Med 16(1):183–190. 10.1007/s41999-024-01103-w 39578318 10.1007/s41999-024-01103-w

[14] Pang J, Wu M, Liu X, Gao K, Liu Y, Zhang Y, Zhang E, Zhang T (2021) Age-related changes in shear wave elastography parameters of the gastrocnemius muscle in association with physical performance in healthy adults. Gerontology 67(3):306–313. 10.1159/000512386 33735906 10.1159/000512386

[15] Tang X, Huang L, Yue J, Qiu L (2025) Dynamic shear wave elastography for the flexor digitorum superficialis: the correlation with physical performance in hospitalized older adults. J Biomech 186:112712. 10.1016/j.jbiomech.2025.112712 40305910 10.1016/j.jbiomech.2025.112712

[16] Cui XW, Li KN, Yi AJ, Wang B, Wei Q, Wu GG, Dietrich CF (2022) Ultrasound elastography. Endosc Ultrasound 11(4):252–274. 10.4103/eus-d-21-00151 35532576 10.4103/EUS-D-21-00151 PMC9526103

[17] Taş S, Onur MR, Yılmaz S, Soylu AR, Korkusuz F (2017) Shear wave elastography is a reliable and repeatable method for measuring the elastic modulus of the Rectus Femoris muscle and Patellar tendon. J Ultrasound Med 36(3):565–570. 10.7863/ultra.16.03032 28108983 10.7863/ultra.16.03032

[18] Hansen JSBK, Andersen-Ranberg K (2023) Assessment of sarcopenia by ultrasound a feasibility study in acutely admitted Danish geriatric inpatients. Eur J Geriatr Gerontol 5(3):196–202. 10.4274/ejgg.galenos.2023.2022-9-4

[19] Manuel Montero-Odasso, Nathalie van der Velde, Finbarr C Martin, Mirko Petrovic, Maw Pin Tan, Jesper Ryg, Sara Aguilar-Navarro, Neil B Alexander, Clemens Becker, Hubert Blain, Robbie Bourke, Ian D Cameron, Richard Camicioli, Lindy Clemson, Jacqueline Close, Kim Delbaere, Leilei Duan, Gustavo Duque, Suzanne M Dyer, Ellen Freiberger, David A Ganz, Fernando Gómez, Jeffrey M Hausdorff, David B Hogan, Susan M W Hunter, Jose R Jauregui, Nellie Kamkar, Rose-Anne Kenny, Sarah E Lamb, Nancy K Latham, Lewis A Lipsitz, Teresa Liu-Ambrose, Pip Logan, Stephen R Lord, Louise Mallet, David Marsh, Koen Milisen, Rogelio Moctezuma- Gallegos, Meg E Morris, Alice Nieuwboer, Monica R Perracini, Frederico Pieruccini-Faria, Alison Pighills, Catherine Said, Ervin Sejdic, Catherine Sherrington, Dawn A Skelton, Sabestina Dsouza, Mark Speechley, Susan Stark, Chris Todd, Bruce R Troen, Tischa van der Cammen, Joe Verghese, Ellen Vlaeyen, Jennifer A Watt, Tahir Masud (2022) the Task Force on Global Guidelines for Falls in Older Adults , World guidelines for falls prevention and management for older adults: a global initiative, Age and Ageing, 51(9):afac205. 10.1093/ageing/afac205 PMC9523684 36178003

[20] Charlson ME, Pompei P, Ales KL, MacKenzie CR (1987) A new method of classifying prognostic comorbidity in longitudinal studies: development and validation. J Chronic Dis 40(5):373–383. 10.1016/0021-9681(87)90171-8 3558716 10.1016/0021-9681(87)90171-8

[21] Charlson ME, Carrozzino D, Guidi J, Patierno C (2022) Charlson comorbidity index: a critical review of clinimetric properties. Psychother Psychosom 91(1):8–35. 10.1159/000521288 34991091 10.1159/000521288

[22] Rockwood K, Song X, MacKnight C, Bergman H, Hogan DB, McDowell I, Mitnitski A (2005) A global clinical measure of fitness and frailty in elderly people. CMAJ 173(5):489–495. 10.1503/cmaj.050051 16129869 10.1503/cmaj.050051 PMC1188185

[23] Rockwood K, Theou O (2020) Using the clinical frailty scale in allocating scarce health care resources. Can Geriatr J 23(3):210–215. 10.5770/cgj.23.463 32904824 10.5770/cgj.23.463 PMC7458601

[24] Barbosa-Silva TG, Gonzalez MC, Bielemann RM, Santos LP, Costa CDS, Menezes AMB (2021) 2 + 2 (+ 2) = 4: a new approach for appendicular muscle mass assessment by ultrasound. Nutrition 83:111056. 10.1016/j.nut.2020.111056 33348110 10.1016/j.nut.2020.111056

[25] Guralnik JM, Simonsick EM, Ferrucci L, Glynn RJ, Berkman LF, Blazer DG, Scherr PA, Wallace RB (1994) A short physical performance battery assessing lower extremity function: association with self-reported disability and prediction of mortality and nursing home admission. J Gerontol 49(2):M85-94. 10.1093/geronj/49.2.m85 8126356 10.1093/geronj/49.2.m85

[26] Motl RW, Learmonth YC, Wójcicki TR, Fanning J, Hubbard EA, Kinnett-Hopkins D, Roberts SA, McAuley E (2015) Preliminary validation of the short physical performance battery in older adults with multiple sclerosis: secondary data analysis. BMC Geriatr 15:157. 10.1186/s12877-015-0156-3 26630923 10.1186/s12877-015-0156-3 PMC4668658

[27] Guralnik JM, Ferrucci L, Pieper CF, Leveille SG, Markides KS, Ostir GV, Studenski S, Berkman LF, Wallace RB (2000) Lower extremity function and subsequent disability: consistency across studies, predictive models, and value of gait speed alone compared with the short physical performance battery. J Gerontol A Biol Sci Med Sci 55(4):M221–M231. 10.1093/gerona/55.4.m221 10811152 10.1093/gerona/55.4.m221 PMC12149745

[28] Welch SA, Ward RE, Beauchamp MK, Leveille SG, Travison T, Bean JF (2021) The short physical performance battery (SPPB): a quick and useful tool for fall risk stratification among older primary care patients. J Am Med Dir Assoc 22(8):1646–1651. 10.1016/j.jamda.2020.09.038 33191134 10.1016/j.jamda.2020.09.038 PMC8113335

[29] Pavasini R, Guralnik J, Brown JC, di Bari M, Cesari M, Landi F, Vaes B, Legrand D, Verghese J, Wang C, Stenholm S, Ferrucci L, Lai JC, Bartes AA, Espaulella J, Ferrer M, Lim JY, Ensrud KE, Cawthon P, Turusheva A, Frolova E, Rolland Y, Lauwers V, Corsonello A, Kirk GD, Ferrari R, Volpato S, Campo G (2016) Short physical performance battery and all-cause mortality: systematic review and meta-analysis. BMC Med 14(1):215. 10.1186/s12916-016-0763-7 28003033 10.1186/s12916-016-0763-7 PMC5178082

[30] Roberts HC, Denison HJ, Martin HJ, Patel HP, Syddall H, Cooper C, Sayer AA (2011) A review of the measurement of grip strength in clinical and epidemiological studies: towards a standardised approach. Age Ageing 40(4):423–429. 10.1093/ageing/afr051 21624928 10.1093/ageing/afr051

[31] Rolsted SK, Andersen KD, Dandanell G, Dall CH, Zilmer CK, Bülow K, Kristensen MT (2024) Comparison of two electronic dynamometers for measuring handgrip strength. Hand Surg Rehabil 43(3):101692. 10.1016/j.hansur.2024.101692 38705572 10.1016/j.hansur.2024.101692

[32] Alfuraih AM, O’Connor P, Hensor E, Tan AL, Emery P, Wakefield RJ (2018) The effect of unit, depth, and probe load on the reliability of muscle shear wave elastography: variables affecting reliability of SWE. J Clin Ultrasound 46(2):108–115. 10.1002/jcu.22534 28990683 10.1002/jcu.22534

[33] Appeadu MK, Bordoni B (2023). Falls and fall prevention in older adults In: StatPearls (Eds) StatPearls [Internet]. StatPearls Publishing, Treasure Island (FL). 32809596

[34] Nielsen BR, Andersen HE, Hovind P, Jørgensen NR, Schwarz P, Kristensen SH, Suetta C (2024) Sarcopenia and self-reported markers of physical frailty in patients with osteoporosis. Arch Osteoporos 19(1):77. 10.1007/s11657-024-01437-9 39152303 10.1007/s11657-024-01437-9 PMC11329389

[35] Saito A, Wakasa M, Kimoto M, Ishikawa T, Tsugaruya M, Kume Y, Okada K (2019) Age-related changes in muscle elasticity and thickness of the lower extremities are associated with physical functions among community-dwelling older women. Geriatr Gerontol Int 19(1):61–65. 10.1111/ggi.13567 30556237 10.1111/ggi.13567

[36] Akagi R, Yamashita Y, Ueyasu Y (2015) Age-related differences in muscle shear moduli in the lower extremity. Ultrasound Med Biol 41(11):2906–2912. 10.1016/j.ultrasmedbio.2015.07.011 26314496 10.1016/j.ultrasmedbio.2015.07.011

[37] Alfuraih AM, Tan AL, O’Connor P, Emery P, Wakefield RJ (2019) The effect of ageing on shear wave elastography muscle stiffness in adults. Aging Clin Exp Res 31(12):1755–1763. 10.1007/s40520-019-01139-0 30762201 10.1007/s40520-019-01139-0 PMC6825644

[38] Şendur HN, Cindil E, Cerit MN, Kılıç P, Gültekin I, Oktar S (2020) Evaluation of effects of aging on skeletal muscle elasticity using shear wave elastography. Eur J Radiol 128:109038. 10.1016/j.ejrad.2020.109038 32422550 10.1016/j.ejrad.2020.109038

[39] Wang Z, Xu Z, Zhong H, Zheng X, Yan L, Lyu G (2024) Establishment and validation of a predictive model for sarcopenia based on 2-D ultrasound and shear wave elastography in the medial gastrocnemius muscle. Ultrasound Med Biol 50(9):1299–1307 38969525 10.1016/j.ultrasmedbio.2024.04.012

[40] Cassel C, Fulmer T (2022) Achieving diagnostic excellence for older patients. JAMA 327(10):919–920. 10.1001/jama.2022.1813 35175301 10.1001/jama.2022.1813

[41] Johnson DC, Elmore JG (2018) Where there is smoke, there is not always fire. Cancer 124(11):2276–2277. 10.1002/cncr.31267 29682726 10.1002/cncr.31267

[42] Bonk J (2010) A road map for the recruitment and retention of older adult participants for longitudinal studies. J Am Geriatr Soc 58(s2):S303–S307 21029058 10.1111/j.1532-5415.2010.02937.x

[43] Koo TK, Li MY (2016) A guideline of selecting and reporting intraclass correlation coefficients for reliability research. J Chiropr Med 15(2):155–163. 10.1016/j.jcm.2016.02.012 27330520 10.1016/j.jcm.2016.02.012 PMC4913118

