# Supplementary material for: Shear-wave elastography for muscle assessment in geriatric outpatients at increased risk of falling
Source: Eur Geriatr Med. 2025 Oct 23;17(1):31–41. doi: 10.1007/s41999-025-01333-6 (PMC12946335; doi:10.1007/s41999-025-01333-6)
Supplement: Supplementary file 1 — Supplementary file1 (DOCX 307 KB) [file 41999_2025_1333_MOESM1_ESM.docx]

**Supplementary material:**


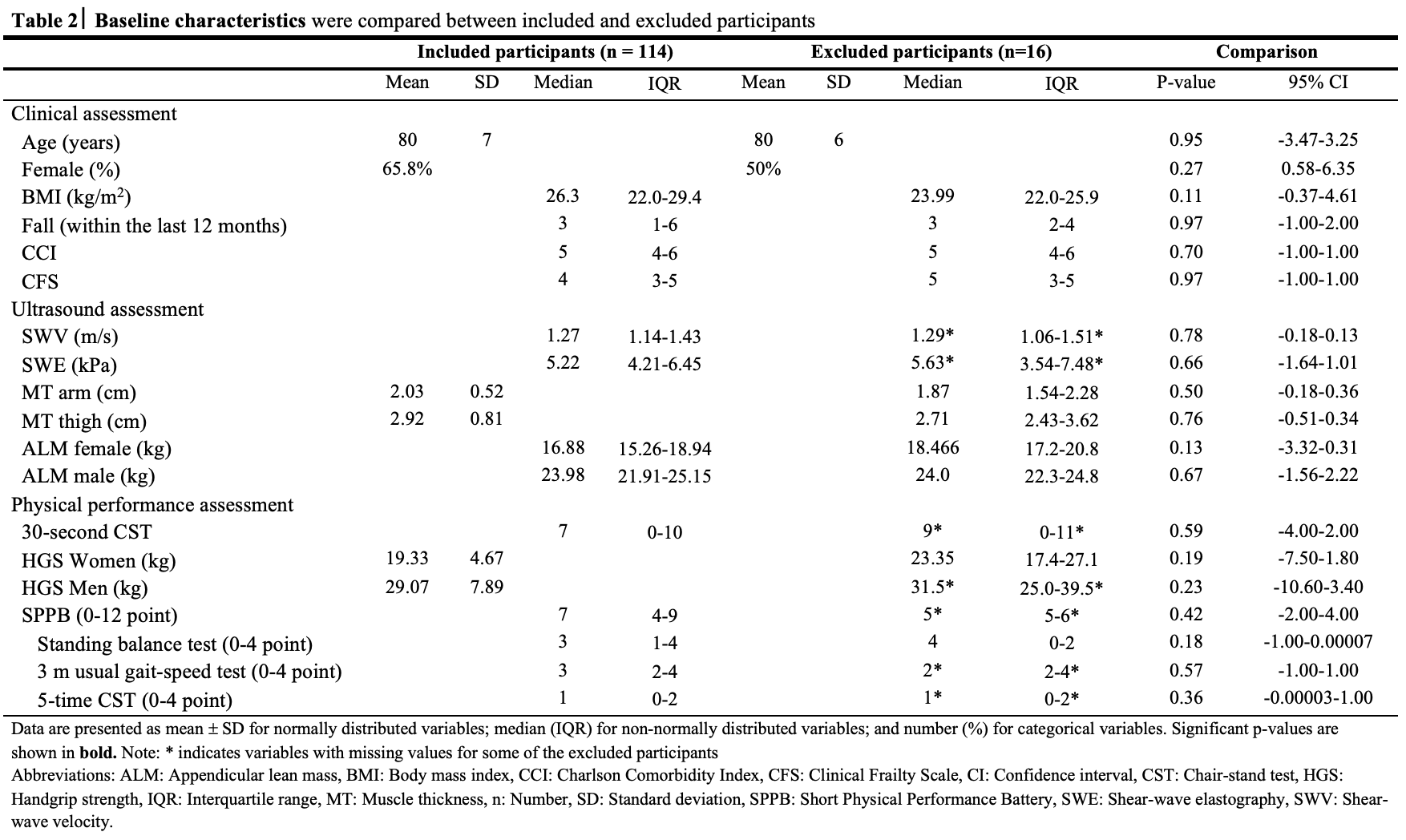
**Table 1: Baseline characteristics comparison between excluded and included participants:**
